# Examining Different Factors in Web-Based Patients’ Decision-Making Process: Systematic Review on Digital Platforms for Clinical Decision Support System

**DOI:** 10.3390/ijerph182111226

**Published:** 2021-10-26

**Authors:** Adnan Muhammad Shah, Wazir Muhammad, Kangyoon Lee, Rizwan Ali Naqvi

**Affiliations:** 1 Department of Computing Engineering, Gachon University, Seoul 13120, Korea; 2 Department of Physics, Charles E. Schmidt College of Science, Florida Atlantic University, Boca Raton, FL 33431-0991, USA; adnanmuhammadsha@fau.edu (A.M.S.); wmuhammad@fau.edu (W.M.); 3 Department of Management Sciences, Shaheed Zulfikar Ali Bhutto Institute of Science and Technology, Islamabad 44320, Pakistan; 4 Department of Unmanned Vehicle Engineering, Sejong University, Seoul 05006, Korea; rizwanali@sejong.ac.kr

**Keywords:** systematic review, physician rating websites, online physician reviews, clinical decision support system

## Abstract

(1) Background: The appearance of physician rating websites (PRWs) has raised researchers’ interest in the online healthcare field, particularly how users consume information available on PRWs in terms of online physician reviews and providers’ information in their decision-making process. The aim of this study is to consistently review the early scientific literature related to digital healthcare platforms, summarize key findings and study features, identify literature deficiencies, and suggest digital solutions for future research. (2) Methods: A systematic literature review using key databases was conducted to search published articles between 2010 and 2020 and identified 52 papers that focused on PRWs, different signals in the form of PRWs’ features, the findings of these studies, and peer-reviewed articles. The research features and main findings are reported in tables and figures. (3) Results: The review of 52 papers identified 22 articles for online reputation, 15 for service popularity, 16 for linguistic features, 15 for doctor–patient concordance, 7 for offline reputation, and 11 for trustworthiness signals. Out of 52 studies, 75% used quantitative techniques, 12% employed qualitative techniques, and 13% were mixed-methods investigations. The majority of studies retrieved larger datasets using machine learning techniques (44/52). These studies were mostly conducted in China (38), the United States (9), and Europe (3). The majority of signals were positively related to the clinical outcomes. Few studies used conventional surveys of patient treatment experience (5, 9.61%), and few used panel data (9, 17%). These studies found a high degree of correlation between these signals with clinical outcomes. (4) Conclusions: PRWs contain valuable signals that provide insights into the service quality and patient treatment choice, yet it has not been extensively used for evaluating the quality of care. This study offers implications for researchers to consider digital solutions such as advanced machine learning and data mining techniques to test hypotheses regarding a variety of signals on PRWs for clinical decision-making.

## 1. Introduction

The association between technological advancements and social changes resulted in the development of physician rating websites (PRWs). This novelty was a result of the emergence and rapid growth of the internet [1]. PRWs offer a unique source of information about healthcare service quality from the patients’ viewpoint. PRWs offer patients an opportunity to rate the quality of service while interacting with the physician. Patients may post ratings or write comments on their experience with a physician or read an assessment for peer patients before choosing a physician [2]. Physicians perceive these PRWs as important because patients’ perceptions of healthcare quality are publicly available. This evidence significantly enhances the relevance of patient satisfaction to create positive word of mouth (WOM). In contrast, reviews posted by patients on PRWs provide recommendations for strengthening and improving overall satisfaction with physicians’ quality of care [3].

Recently, there has been growing interest in PRW usage, which has become a part of life for many of us. The Internet has enabled the massive growth of PRWs. PRWs are organized in a similar manner to other rating sites (for instance, tourism, hotels, or restaurants). Rating sites for search (products) and experience goods (hotels and restaurants) have already become popular, but this is a fairly new internet-based rating platform in the medical domain. A variety of investigations were performed using different PRWs in different countries, such as RateMDs [4], Healthgrades [5], and Vitals [6] in the U.S.; Haodf [7] in China; Jameda [8] and Weisse Liste [3] in Germany, and Iwantgreatcare [9] in the U.K. The culture of reviewing in healthcare developed in parallel with a shift in the patient–physician relationship. The notion of reviewing in healthcare results in a change in the doctor–patient (D–P) relationship. The conventional bond between doctor and patient has changed into a patient-centered approach; as a result, patients play a more authoritative role in their health decision-making [10].

The PRWs tend to offer several benefits to patients. They can provide valuable information to them, help patients to search for physicians with high technical skills, and assist them in their choice to select the best suitable physician. PRWs can also boost treatment quality and foster trustworthy relationships between doctors and patients [11]. Patients are more frequently dependent on PRWs if the information that they search is unique to their requirements. There are also some drawbacks of PRWs: physicians are afraid that PRWs promote negative feedback [7]. However, a study of online physician reviews (OPRs) found that these reviews were overwhelmingly positive. Moreover, very few reviews raise questions about the representativeness of decisions and scientific validity, particularly by healthcare providers and health organizations [12]. PRWs are effective if patients search for systemic details (e.g., service availability, operating hours, and place of office) rather than process or outcome aspects. In terms of outcome measures, PRWs are capable of providing information, but PRWs can cause confusion and pose risks to an evaluated individual regarding outcome measures [13].

In our context, healthcare providers may not serve consumers’ best interests and benefit from information asymmetry. For example, healthcare providers who run their own clinics or hospitals may refer patients for unnecessary care to such facilities and benefit financially from doing so. Therefore, to reduce information asymmetry, PRWs offer patients different signals, which help them in their choice toward a specific physician. In healthcare, the role of the signaling mechanism becomes important for the following reasons. First, by delivering informative signals, a good doctor will credibly pass on the medical service quality to patients [14]. Second, in PRWs, patients can put pressure on their physicians by possibly obtaining a second opinion. In the context of online healthcare, physicians send signals about the quality of service to their patients. Upon receiving this information, patients can change their decision on physicians’ quality of service and change their physician choice [14]. If information is asymmetric, patients and doctors need to send meaningful signals to inform these patients in an efficient and reliable manner that is under-informed about the particular doctor’s healthcare quality. The selection of appropriate signals in the PRWs is vital to these rating websites’ success because different signals can express different types of information and eventually lead to uneven outcomes.

The growing importance of PRWs in the patients’ decision-making process has resulted in an increasing number of research studies on PRWs [15,16]. Some researchers have argued in favor of giving PRWs more systematic values [1,17]. Others have measured and reviewed public perceptions and use of PRWs in evaluating the quality of PRWs [2,18]. Researchers found that there was limited research on the usage of PRWs [10,15]. To the best of our knowledge, there is no systematic review of the different signaling mechanisms (online and offline signals) generated by the market and sellers on PRWs. Therefore, we performed a systematic analysis to summarize the study features, research design, analytical methods, and current PRW studies’ main findings. We have shown the different PRW research patterns, identified literature shortcomings, and made recommendations for potential study.

## 2. Methods

The current research consists of a systematic and detailed literature analysis on different signals related to PRWs in healthcare. We adopt the recommendations suggested by Hong, Liang, Radcliff, Wigfall, and Street [15]. A systematic analysis of the literature has a threefold objective: review planning, to carry out the review, and report the critical findings. This leads to an in-depth understanding based on the theoretical review of existing research. We started to design the systematic review performed in this paper in May 2019. The search for publications was performed from November 2019, with several rounds of refinements and improvements.

### 2.1. Planning the Systematic Review

The review protocol was developed based on the recommendations of the Preferred Reporting Items for Systematic Review and Meta-Analysis Protocols statement [19]. The systematic review will also adhere to the recommendations of the Preferred Reporting Items for Systematic Reviews and Meta-Analyses (PRISMA) guidelines [20]. This protocol is under review for registration with the International Prospective Register of Systematic Reviews.

We designed the review by putting forward research questions related to our research goals. We defined the search strategy, search strings, and inclusion/exclusion criteria. We specified research questions, search strategy, selection criteria for inclusion and exclusion, and findings. A brief explanation of these issues is presented below.

### 2.2. Research Questions

In this paper, the following questions needed to be addressed:

**RQ1**. How can the interaction of online and offline signal transmission on PRWs provide benefits regarding patients’ choice for a health consultation?

**RQ2**. What are the reviewed studies’ dynamics and analytical approaches involved in patients’ decision-making process?

### 2.3. Search Strategy and Criteria

To carry out our research, we followed the guidelines provided by the Preferred Reporting Items for Systematic Reviews and Meta-Analysis (PRISMA) [19]. Next, the search strategy was then used to evaluate all the available relevant studies to answer the research questions. In this paper, we retrieved relevant studies published between 2010 and 2020 from the major databases, digital libraries, and published proceedings, including PubMed, EMBASE, Google Scholar, Scopus, Web of Science (Clarivate Analytics), Science Direct, Emerald, Taylor & Francis, Springer, Sage, ACM, Wiley, and IEEE, in January 2021 (refer to Table 1). Keywords from previously published studies, including health rating platforms, physician rating websites, review sites, online reviews, online physician reviews, online ratings, patient online reviews, healthcare quality, e-health, and digital health, were used in the search terms. A Boolean operator (AND) was applied between the keywords that the authors used for searching different databases and the search for matching keywords in paper titles or abstracts. Finally, we applied HistCite software (A software program that makes bibliometric analysis and visualization tasks easier for researchers) on the collected literature.

**Figure 1:**
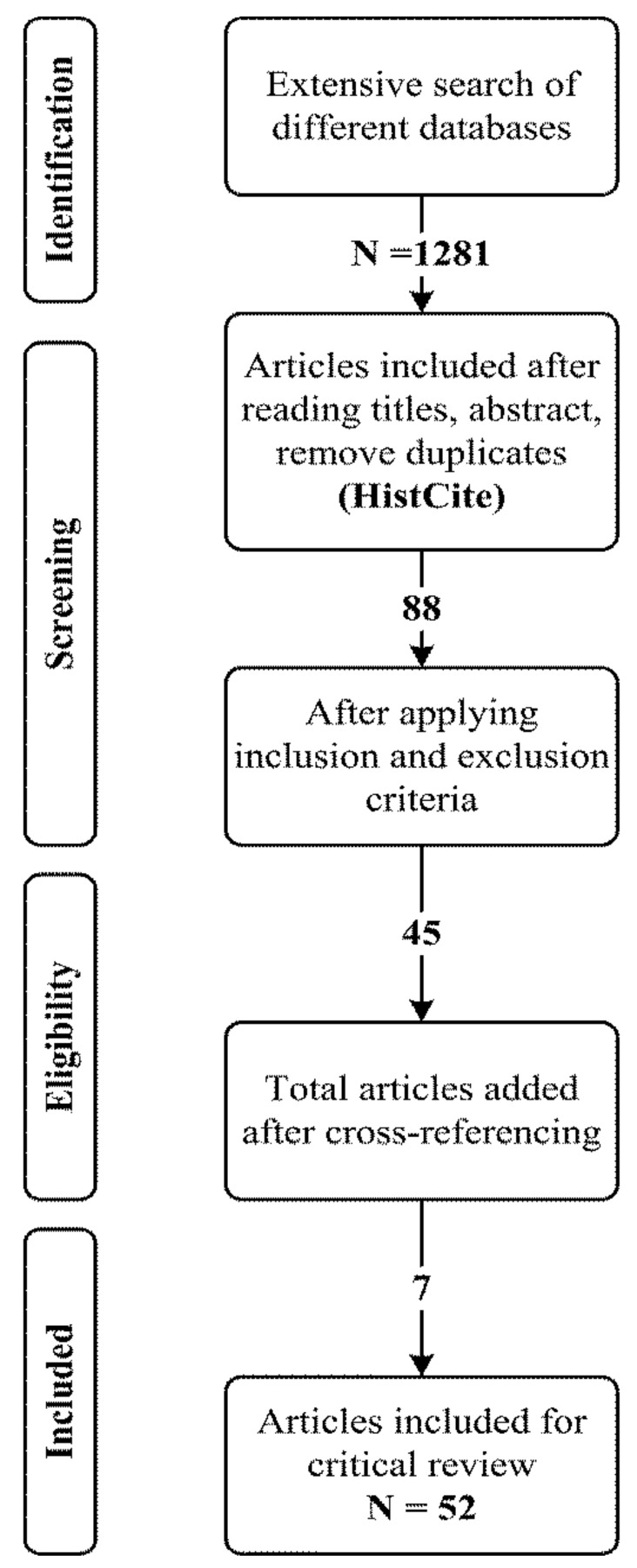
Stages of the literature search process.

Figure 1 shows the initial search phase using the above keywords, which retrieved 1281 articles. After reviewing the titles and abstracts, based on the snowballing technique, 10 articles were found as duplicates and were removed from our list. Hence, after the deletion of duplicates, the total number of remaining articles was 88. The remaining articles were reviewed against inclusion and exclusion and quality evaluation criteria (see Section 2.4 and Section 2.5). Hence, 45 publications were found to satisfy the criteria. Next, we performed the reference searches of 45 papers; hence, 7 articles were added from cross-referencing. Finally, we identified a total of 52 articles to include in the review (see Table 2). Articles documenting results based on a similar data source, the same research design, and research questions were considered as one study.

### 2.4. Inclusion and Exclusion Criteria

The inclusion and exclusion criteria of the retrieved studies are listed in Table 3. The quality criterion used by other researchers was used in this systematic analysis [21,22,23,24]. One of the essential criteria to be tested for the inclusion and exclusion of studies is the methodological quality assessment. The quality of each published paper was evaluated using the following three indicators.

- First, has the Web of science or Scopus database indexed the selected papers?
- Second, is the study aim/objective clear?
- Third, is the research context dealt with well?
- Finally, the last question helped us determine whether the research findings were sufficient for our research purpose.

### 2.5. Quality Assessment

The evaluation of research quality can be used to direct the interpretation of the synthesis [25]. The quality criterion, as employed by others, was used for this systematic analysis [22,23]. The quality of each study approved was assessed in accordance with the requirements outlined in Section 2.4, as shown in Table 4. With the first criterion (C1), we assessed whether researchers clearly addressed the study aim/objective. The majority of the research (88%) gave a favorable response to this question. To find out if the research context had been adequately addressed and articulated, we used criterion (C2). Overall, 92% of the studies had a favorable response to this question. The final question helped us to determine whether the research findings were sufficient for our research purpose. For the heuristic grades for the quality measurement (C3), 2 reviewers examined and analyzed all of the studies (AS and WM), and a third independent expert (RN) resolved conflicts between the 2 independent reviewers. The quality score of 52 included papers evaluated by 3 reviewers is shown in Table 5.

### 2.6. Data Extraction and Synthesis

At this point in the analysis, the selected papers were synthesized and classified in accordance with the scope of their various characteristics in the PRW, which affect the patients’ decision-making process. In order to address research questions, the relevant data from 52 papers were collected, analyzed, and summarized. Two co-authors of this study performed content analysis by reviewing all selected articles included in a review [23]. Both co-authors developed a form for the recording of thoughts, concepts, contributions, and findings for each of the 52 studies; subsequent higher-order analysis was assured by using this form. The following data were extracted from each publication: signals used on the PRWs, time and place of the study, signal transmission across different disease specialties, number of reviews by different PRWs, study design and technological roadmap adopted, and key findings, The inter-rater agreement between two researchers was calculated by using the Cohen kappa and was 0.83, indicating good agreement. The researchers then listed the main findings from each article and addressed their differences until an agreement was reached.

## 3. Findings

This section presents the findings of our review in the context of our research questions.

### 3.1. Overview of Publications

**Figure 2:**
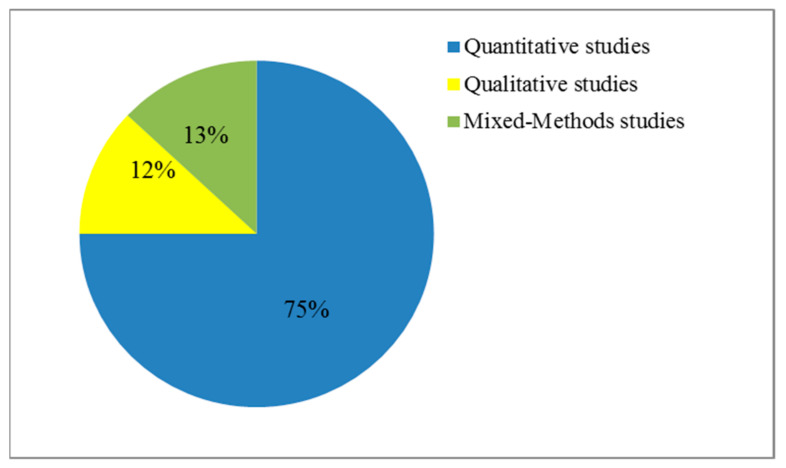
Categorization of studies.

As indicated in the previous section, we identified 52 articles. Out of the 52 studies, around 16% (8 of them) were published in conferences and 84% (44 of them) in journals. The distribution of the included articles, which were published from 2010 to 2020, is shown in Figure 2. Figure 2 indicates that 75% used quantitative techniques, 12% employed qualitative techniques, and 13% were mixed-methods investigations.

### 3.2. Evaluation Criteria and Statistical Analysis of the Signaling Mechanism

The signaling mechanisms include online and offline signals produced by different online and offline sources. Online signals are generally produced by both marketers and sellers, whereas sellers are responsible for generating offline signals. The effectiveness of these signals on patients’ choice is evaluated by the adjusted *R*^2^, standardized coefficients (*β*), and significance value.

### 3.3. RQ1. How Can the Interaction of Online and Offline Signal Transmission on PRWs Provide Benefits Regarding Patients’ Choice for a Health Consultation?

Information asymmetry is most likely to exist between sellers and consumers in online markets [26]. It may cause sellers to influence the consumers’ purchase behaviors, the seller’s performance, and boost fraudulent activities. These arguments are especially true in the case of credence goods, such as online healthcare services. As earlier discussion stated, the services offered in the online healthcare market are considered credence goods such that doctors already know more about their quality of services and patients’ health status [27]; moreover, information asymmetry is more severe in the online healthcare environment.

#### 3.3.1. Physician’s Online Reputation

The physician’s online reputation refers to patients’ perceptions regarding the physician’s online assessment after each interaction [28]. To select a competent physician, health consumers take suggestions from their family members and friends to obtain WOM information. Online reputation is a part of this eWOM information. PRWs allow patients to review the abundant information about various physicians and then use this eWOM information for their health consultation; the information contains a physician’s online reputation for medical services. Out of 52 studies, 22 (22/52, 42%) reported online reputation as a signal of a physician’s healthcare quality [5,28,29,30,31,32,33,34,35,36,37,38,39,40,41,42,43,44,45,46,47,48]. The existing WOM literature validates eWOM as a valuable and efficient channel for disseminating information regarding providers’ reputation to consumers.

In this context, Yang, Guo, Wu, and Ju [47] indicated that online reputation in the form of ratings and experience from others could be used to reduce information asymmetry, which further helps in patients’ decision-making process. Furthermore, Li, Tang, Yen David, and Liu [38] investigated the positive impact of online reputation on the number of physician bookings.

Hence, the above arguments suggest that ratings from peer consumers influence a patient’s choice; healthcare providers could design some programs in encouraging users to rate their doctors and write OPRs in order to fulfill their needs, evaluate healthcare quality, and enhance users’ collaborations in PRWs.

#### 3.3.2. Physician’s Online Effort

On PRWs, the physician conducts online activities to convey quality information in the form of benevolence actions. These activities are linked to the physician’s online efforts, referring to “the amount of energy ‘spent’ by a doctor on an act per unit of time” [38]. A total of 14 (27%) studies were related to physicians’ effort online [14,28,31,32,38,49,50,51,52,53,54,55,56,57]. For example, Liang et al. [58] found that the online efforts and reputations of physicians have a significant impact on the number of new patients. Li, Tang, Jiang, Yen, and Liu [49] and Li, Tang, Yen David, and Liu [38] reported that knowledge contributions as online effort are significantly related to patients’ choice toward a specific physician.

#### 3.3.3. Service Popularity

Popularity is the degree to which many people are familiar with a product, and they assess it from the perspective of quantity. On PRWs, physicians use multiple channels to signal their popularity [45]. The use of online healthcare services can reveal a physician’s service popularity and decrease the perceived risk of low-quality services. For instance, Gao et al. [59] found that service popularity is an important factor in shaping patients’ choices. In our systematic review, 15 (29%) studies reported physician popularity as an indicator of patient decision-making [31,38,45,50,51,53,59,60,61,62,63,64,65,66,67]. Thus, the role of e-health to measure the popularity of provider services has grown and become more popular in recent years.

#### 3.3.4. Linguistic Signaling

PRWs contain a great deal of linguistic signals, a valuable resource for people seeking health information and social support. Information quality as a linguistic signal refers to the message’s persuasive strength, which is commonly measured in terms of its relevance, timeliness, accuracy, and comprehensiveness [52]. Reviews posted by different users are always different in length, accuracy, comprehensiveness, tone, domain, and even logic. Therefore, in an online environment, users perceive the information regarding a particular activity in which they are engaged, considering their expectations and requirements. Most of the literature employed “argument quality or information quality” to measure the information quality as a predictor of users’ behavior in the healthcare domain. In this literature review, we found 17 (32.69%) articles that indicated the impact of linguistic signals on patients’ behavior [52,67,68,69,70,71,72,73,74,75,76,77,78,79,80,81,82].

#### 3.3.5. Doctor–Patient Concordance Signals

D–P concordance refers to the agreement between a patient and his/her physician regarding the diagnosis and treatment of a condition [83]. Prior studies discussed the concept of D–P concordance, and Banerjee and Sanyal [84] found that strong D–P concordance (agreement) signals the physician’s better trust, which in turn leads to patient satisfaction. According to Audrain-Pontevia et al. [85], patient empowerment and patient commitment are significant indicators of D–P concordance and patient compliance. These findings highlighted the improvement in D–P concordance. In the comprehensive analysis of the previous literature, we identified 15 (29%) articles related to D–P interaction, concordance, and their association with patients’ behavior [29,73,83,84,85,86,87,88,89,90,91,92,93,94,95].

#### 3.3.6. Physician’s Offline Reputation

Most sellers launched online platforms to extend their current traditional offline channels to survive in the competitive market. In this way, users are gradually shifting from single-channel users to multi-channel users through channel extension.

In our research context, patients consume the commodity (healthcare) provided by the physicians. This means that the offline reputation of a (individual) physician is essential to a patient in making a decision to determine the physician’s healthcare quality. The offline reputation refers to the physician’s medical status (his/her title) assignment by the government according to the physician’s competency and ability. A physician with a high reputation transmits the credibility signal to the receiver. For instance, Liu, Guo, Wu, and Wu [29] found the positive impact of a physician’s offline reputation on patients’ choice. In the patients’ health consultation decision, signals such as status affect a provider’s reputation. In this literature review, seven (13%) studies focused on the effect of physicians’ offline reputation on patients’ decision-making process [28,38,53,96,97,98,99].

#### 3.3.7. Physician Trustworthiness Signals

PRWs are a means for physicians to signal their trustworthiness. Trustworthiness is defined as the provider’s ability (credibility) and willingness to provide support and advice in the consumer’s best interest [100]. In other words, trust is a belief and expectations about an exchange partner’s credibility [5]. Researchers have claimed that a patient’s trust in a physician is important for the fairly unknown e-health relationship between physician and patient. Researchers have claimed medical boards’ data and other web sources as a measurement of credibility. They suggested that when linking ratings with the state medical board data, board-certified, highly experienced doctors, and doctors who graduated from higher-ranked schools had superior ratings [5]. It has also been found that a greater number of unsatisfactory ratings was associated with a history of malpractice claims or medical board actions and sanctions identified on the doctor’s page [101]. Moreover, the more awards awarded to a physician, the more credible s/he was perceived to be [5]. In this review, we found 11 (21%) studies that reported on physician credibility and its impact on patients’ decision-making [5,12,40,44,78,100,102,103,104,105,106].

### 3.4. RQ2. What Are the Reviewed Studies’ Dynamics and Analytical Approaches Involved in Patients’ Decision-Making Process?

Descriptive statistics were performed to capture the data collection procedure, variables, the country where the research was conducted, research methods, and findings.

#### 3.4.1. Time and Place of the Study

**Figure 3:**
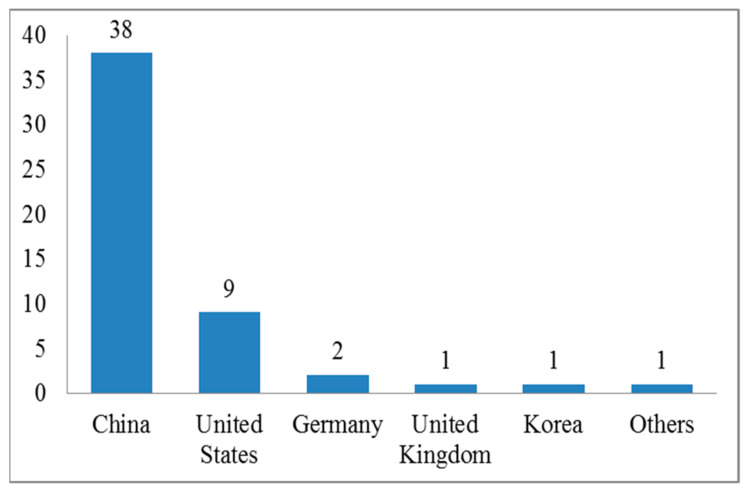
Country-wise location of studies.

Although PRWs have been available for over two decades, the earliest research on different signal transmission on PRWs was published in 2010 [107], and the majority of studies were conducted (61/63, 96.8 per cent) after 2015. As shown in Figure 3, most of the studies belonged to China (38) [7,15,28,30,31,32,33,34,37,38,40,44,45,46,47,48,49,50,51,52,53,54,56,60,61,63,65,73,81,104,106,108,109,110,111,112,113] followed by the U.S. (9) [4,5,14,34,35,67,78,99,114], Germany [3,115] (2), the U.K. (1) [9], Korea (1) [36], and others (1) [116].

#### 3.4.2. Physician Rating Websites

**Figure 4:**
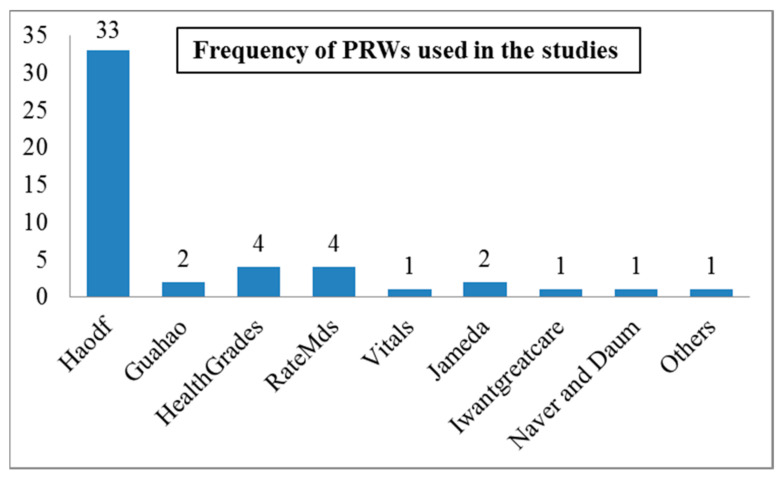
Physician rating websites used in studies.

The majority of studies (41/52, 78.84%) considered a single PRW for data analysis using different signals. In contrast, several studies used multiple PRWs for data analysis (6/52, 11.53%), and the rest of the studies performed primary data analysis using survey questionnaires, etc. (5/52, 9.62%). The PRWs used in these investigations differed across different countries. For example, in China, a large number of signal transmissions were accrued on the most popular specialized PRWs on Haodf and Guahao. Next, RateMds, HealthGrades, and Vitals [4,5,6,117] were the most popular and frequently used rating platforms in the U.S., whereas the Jameda [3,115] and Iwantgreatcare (a subsidiary of the National Health System Choices website) [9] were Germany’s and the U.K.’s most popular PRWs, respectively (see Figure 4).

#### 3.4.3. Signal Transmission across Different Contexts and Disease Specialties

Out of the 52 studies, one (1/52, 1.92%) reported different signals in a hospital context, including hospitals, clinics, emergency departments, and nursing homes [36], and 51 (51/52, 98.02%) were focused on physicians from different disease specialties. Of the 51 studies that reported different signals of physicians, nine (9/51, 17.65%) involved different types of physicians (general practitioners and specialists), one study (1/51, 1.96%) reported on dentists [8], and the remaining 41 (41/52, 80.39%) were focused on disease specialists, including cardiologists, oncologists, neurologists, orthopedics, pulmonologists, ENTs, endocrinologists, dermatologists, urologists, and Ob/Gyns. Of these 41 studies on disease specialists, 34 (34/41, 82.92%) were focused on multi-specialties.

#### 3.4.4. Number of Reviews by PRWs

The number of physicians reviewed ranged from 512 to 178,740 in these studies. The number of OPRs analyzed ranged from 3000 to 1,274,255. The amount of OPRs involved in the analyses has increased dramatically over the past 10 years. In total, the highest number of reviews was found on Haodf with 56,334 reviews, followed by Guahao with 28,298 reviews, RateMDs with 24,233 reviews, Healthgrades with 19,233 reviews, Vitals with 15,465, Yelp with 11,675 reviews, Jameda with 9876 reviews, and Iwantgreatcare with 7656 reviews. Overall, 347/351 (99.28%) board-certified physicians had been reviewed on at least one of the eight involved websites.

#### 3.4.5. Study Design and Technological Roadmap Adopted

Several articles (5/52, 9.61%) were descriptive. They only described frequency analyses, including the average number of ratings per physician as a proxy of online reputation, the proportions of physicians that had been reviewed online, and the average rating score of OPRs. Usually, research that concentrated on all kinds of disease specialists collected OPRs directly from PRWs without a preselected list of doctors.

A considerable number of articles (39/52, 75%) were quantitative. Articles that focused on healthcare organizations and different specialties identified the provider’s specialty from the perspective of disease mortality from a state disease control and prevention website. In contrast, studies that focused on all types of specialties retrieved data directly from PRWs without considering a list of specialties.

More than six (12%) articles were purely based on qualitative analysis of OPRs. These articles used different computational methods to retrieve major themes from patients’ comments.

A total of seven (13%) articles also analyzed unstructured comments of OPRs along with quantitative analysis using advanced text mining and artificial intelligence techniques such as natural language processing (NLP) and sentic computing models [5,35,52,71,95]. Approximately five (9.61%) articles describing quantitative studies used traditional survey methods to predict patients’ behavior (i.e., clinical outcomes, healthcare quality, and patient satisfaction) [16,73,85,115,118].

The unstructured comments of OPRs were analyzed in a total of 14 (26.92%) articles. Previous research had used the traditional qualitative content analysis to analyze these comments in order to retrieve the key themes in these OPRs [7,37]. More advanced techniques such as NLP have been used in recent articles [4,33,34,35,36,37,112,114]. For example, topic models, such as Latent Dirichlet Allocation, point out the different themes involved in online reviews that may be linked to one of the topics. This has been extensively used to identify topics from unstructured data in different domains, particularly in healthcare. In addition, one article used PRWs from two different countries (China and the United States) for analysis [34].

Finally, a few articles used secondary panel data to conduct analysis (9/52, 17%). Appendix A lists the number of articles focusing on e-health and also provides details about the core components of the PRWs regarding patients’ decision-making toward the physician’s quality of service and explained variable(s).

#### 3.4.6. An Overview of the Findings of Online Physician Reviews

Most patients commented favorably on their physician’s reputation and claimed that they would recommend their physician to their circle of friends and family members. Out of 52 articles, 22 articles (22/52, 42.30%) reported average rating scores of OPRs ranging from 2.81 to 4.62 (with a 5-point rating scale) having a median score of 4 and a mean value of 3.95. The articles that analyzed the patients’ unstructured comments found that these comments covered the different aspects of healthcare, including doctor value, treatment/operational process, doctor attitude, convenient hospital location, disease diagnosis, patient visit process, medical ethics (relational conduct), medical examination, physician knowledge and confidence, parking availability, treatment cost, and physician skills regarding pain control.

#### 3.4.7. Relationship between Signal Transmission and Clinical Outcomes

Appendix A also includes summaries of the relationship between different signaling mechanisms and patients’ choice as clinical outcomes. The majority of the 52 articles on the relationships between signaling mechanisms and patients’ choice reported a positive relationship. For instance, Li, Tang, Yen David, and Liu [38] found a positive relationship between online reputation and patients’ choice of physician selection. In a similar vein, Shah, Yan, Shah, Shah, and Mamirkulova [5] reported a positive association between a physician’s online or offline reputation and patients’ decision-making process for health consultations.

## 4. Discussion

Patients’ decision-making process using different signals on PRWs has increasingly gained attention from various stakeholders in the healthcare industry. This literature review aimed to identify different signaling mechanisms to investigate their impact on patients’ choice toward a particular doctor. The reviewed literature demonstrates several advantages of studying different signals that originate from various sources, such as senders (physicians) and receivers (patients). Generally speaking, this is the first systematic analysis of research on different signaling mechanisms on PRWs. The 52 articles included in this review represent a decade of peer-reviewed publications on PRWs from six countries; the research design and main findings have been summarized.

Research on search and experience goods has received considerable attention from the academic community. The relationship between online signals, offline signals as eWOM, and seller reputation has a significant effect on consumers’ choice [119,120] and has been studied by researchers. A major limitation of this literature is that numerous signals are used to investigate the customer purchase decision-making from a search and experience goods perspective. Credence goods such as healthcare services are different from the aforementioned goods. The quality of healthcare services is difficult to measure for patients, even after they have utilized the services. Therefore, a significant gap exists as patients’ decision-making process using different signals on PRWs has not received substantial research attention in the online healthcare environment.

Our comprehensive analysis of the 52 reviewed articles on PRWs showed that the number of physicians being reviewed constituted a small percentage of the total workforce in healthcare. Overall, the relationships between reputation signals and patients’ choice were positive. Only a few articles compared the associations between different signals on PRWs and the patients’ preference toward a specific physician. These articles showed that online and offline signals were strongly associated with the “patient experience” measured by conventional surveys, quantitative approaches, qualitative approaches, and mixed-methods investigations.

The existing PRW literature indicates a fairly new but rapidly growing field. In comparison with the exponential growth in PRW usage, the number of published articles was limited. Therefore, we offer the following suggestions for possible extensions of PRW research in the future.

First, since the context of the study is the online healthcare industry, services received through offline channels are quite different from online channels. Patients search for physicians and disease information online and book appointments through online channels. They visit offline hospitals to seek treatment for the disease and pay the cost of the medical services. Researchers need to develop new techniques for online information consumption to evaluate the provider’s offline service quality through online information channels.

Second, previous research mainly investigated system quality and the quality of technology used [52] rather than the outcome of a provider’s service. In order to better understand the online service quality, further investigations are required, especially for pure industries such as online healthcare services, which require minimal physical interaction. In these settings, the investigation should focus on the different online signals and offline signals generated by the market and sellers and summarize the main antecedents influencing patients’ choices for treatment decisions.

Third, the patient’s clinical decision-making process is an important issue in healthcare and has gained popularity with the growth of social media websites. In the current literature review, we assume that physicians deliver the product (healthcare) to their patients. This assumption suggests that in evaluating service quality, a physician’s organizational status (i.e., offline reputation and credibility) and online status (i.e., online reputation, online reputation, service popularity, information quality, social influence, and D–P interaction, etc.) about the physician’s clinical quality plays a significant role in the patient’s choice. Furthermore, the physician’s offline status is quite different from offline brand status, as the physician’s offline status may be regarded as the physician’s status in the offline hospital. Nevertheless, the offline brand status is considered an attribute of the product. In addition, regarding consultation decisions in an online environment, the product for sale is the healthcare provider. Existing studies in the field of e-health have discussed the various signals sent by patients [14]; nevertheless, as it is fairly recent, the theoretical distinction has been given less attention to these signals and receiver perception is ignored, which calls for further research.

Fourth, in the previous e-market research, there exists a clear gap regarding the classification of the signals. Based on the existing research on different information channels, there exists scant research that has explored the different types of online and offline signals (reputation signals, trustworthiness signals, service-related signals, linguistic signals, and D–P interaction signals) in a single study. Future research should address all these signals in one study to compare their effects directly due to the specific laboratory conditions and independent study outcomes.

Fifth, in order to examine patient behavior, articles with a systematic design, longitudinal character, and broad samples are required. Because of the availability of broad and heterogeneous online data on the rating sites, PRW studies face challenges in the collection and processing of data. The new methods for web scraping have made it possible to retrieve vast volumes of OPR data efficiently. Advanced computational methods such as machine learning, deep learning, artificial intelligence, and other NLP techniques can be used to accelerate large-scale OPR analysis. Such tools can help researchers to analyze a large quantity of patients in real time in order to investigate previous patients’ opinions about the service quality.

Sixth, most of the current studies concentrate on specialists in high-tier cities based on the highest internet usage, the highest number of physicians with an active board license, and the largest population in the U.S. [5,9,67]; to avoid the sampling bias of patients’ behavior, more studies would be needed to understand other disciplines of healthcare providers and those practices in low-tier cities. There is little or a lack of evidence in research documenting different signals on PRWs, such as nursing homes, public health facilities, and centers for drug treatment.

Finally, in the extant literature, patients’ opinions have been identified explicitly using different machine learning and text mining techniques. However, in everyday life, opinions are expressed implicitly depending on the domain and context. We, therefore, conclude that further work is needed to implicitly analyze opinions to examine patients’ behavior using powerful, intelligent systems.

### 4.1. Implications of the Study

The growing body of PRW-related literature shows its increasing importance in patients’ decision-making process, which offers implications for physicians, patients, PRW developers, and policymakers in both policy and practice.

In particular, the significance of different signals on PRWs should not be underestimated by health providers. Instead, they should consider PRWs’ significance for their “digital branding” and remain conscious of the various signals to prominent PRWs [121]. Physicians may use different signals for patient satisfaction evaluation and determining patient needs. Furthermore, customized and friendly responses to OPRs will boost the positive D-P interaction.

From a consumer viewpoint, patients should realize that only a small number of doctors have been rated online, and the average rating score for a physician may not be adequate to choose a doctor as expected, considering consumers’ propensity to provide reviews on exceptionally favorable or negative encounters [14]. With increasing knowledge about healthcare, we expect that a “market guide” would assist patients in understanding OPRs and making more knowledgeable choices [122].

For PRW developers, as OPRs are often unstructured and the identity of the reviewer cannot be identified, they can take additional social obligations through the introduction of interface components to permit identity authentication, delete offensive or abusive remarks, and help patients in the use of PRWs to prevent fake or mis-information on PRWs [123], which is particularly important in light of the recent COVID-19 crisis [124].

Policymakers still face a major challenge as to whether OPRs can be utilized as an indicator of healthcare quality; policymakers and healthcare providers should recognize and acknowledge their growing importance for patients. The OPRs can reflect immediate feedback from the clinical interactions of patients, their evaluations, and what they really value. Some of the patient experience signals identified by OPR analysis can be applied to support or supplement current healthcare quality measures and to quickly identify perception deficiencies, along with service improvements or other practical quality measures, when necessary [125]. While recognizing the increased weight of OPRs in consumer health conduct and the potential of using OPRs to improve the quality of healthcare, it calls on key stakeholders, including patients, carers, physicians, PRW designers, policymakers, and healthcare service researchers, to engage in discussions and joint efforts to build a positive D-P relationship.

It is necessary to remember any possible biases when evaluating the findings of this study. First, this analysis concentrated on the published studies that focused on PRWs, so the PRW-related results only represented those published studies and not the entire context of PRWs. In view of the large and increasing number of papers written on PRWs, only a small fraction have been researched and reviewed. Second, there are only a few patients who post online reviews and ratings. Younger females, who live in metropolitan areas and spend more time online, are most likely to be these patients. Thus, the current OPRs have a possible bias. Such biases are not designed flaws in performing a systematic review but need preventive measures when interpreting study results.

The fact that users interpret and benefit from such signals available on PRWs containing information about health decision-making contributes to the lengthy search time for patients. This impact will help to improve the patients’ healthcare knowledge [126]. Users faced with meaningful signals spent roughly five fewer minutes while searching for health information online. Researchers and medical practitioners using different signals should be mindful that the structure of signal transmission can be equally as critical as its content in evaluating their impacts on the clinical decision support system [127].

Moreover, signals may also carry possible risks in favor-sensitive decisions. The influence of signaling bias is becoming more and more evident, where signals can supersede decisions relating to risk. Signals are extensively used in patient clinical decision support [128]. It is also possible that signals are being used as clinical decision-making support by other patients, even though they are not declared expressly as decision support [127]. Decision support includes evidence-based resources intended to help patients to make specific healthcare decisions in a value-driven manner [129]. Signals can reduce decision support effectiveness through the presentation of asymmetrical data or by overriding decision-making information through signaler characteristics [130]. For instance, a study conducted by Drewniak, Glässel, Hodel, and Biller-Andorno [127] indicated that clinical decision support systems were more likely to portray patients that were satisfied with the outcome of their treatment decision. This highlights the importance of different signaling mechanisms in PRWs and their impact on patients’ treatment decisions.

### 4.2. Limitations

This study has certain limitations. First, due to the growing literature in this research field, it is possible that some published studies were not included in the review. Second, the keywords used for publication searches might not have been suitable to obtain all studies on PRWs. In the future, more literature will be found on how to improve the usage of PRWs. Finally, since our research was limited to the English-language literature, the review did not include publications written in other languages. Future research might include publications in other languages as well.

## 5. Conclusions

The current peer-reviewed literature on the use of PRWs by health consumers and professionals suggests that PRWs are viewed as valuable knowledge platforms when searching for health information online. This review found that online and offline signals generated by the market and sellers tended to be positive. These signals have a significant and positive impact on patients’ decision-making and clinical decision support system. Findings from this systematic literature review provide insights to guide patients, medical practitioners, and policymakers to assist patients in making more informed decisions and promoting the use of PRWs to enhance the quality of healthcare. The findings of this study call for future research using a large sample size and longitudinal study design.

## Figures and Tables

**Table 1:** Search sources.

Inclusion Criteria	Exclusion Criteria
Electronic databases	PubMed, EMBASE, Google Scholar, Scopus, Web of Science (Clarivate Analytics), Science Direct, Emerald, Taylor & Francis, Springer, Sage, ACM, Wiley, IEEE
Searched items	Journals and conference proceedings
Keywords used	Health rating platforms, physician rating websites, review sites, online reviews, online physician reviews, online ratings, patient online reviews, healthcare quality, e-health, digital health
Searched applied on	Full text to locate publications that fell within the scope of our search and to ensure that we did not overlook those that did not include our search keywords in their titles or abstracts.
Language	English
Study period	January 2010–December 2020

**Table 2:** The number of articles that were filtered based on search terms.

Database	Retrieved	Included
PubMed	75	11
Science Direct	156	10
Emerald	189	2
Taylor & Francis	195	8
Springer	178	7
Sage	165	5
ACM	158	4
Wiley	149	3
IEEE	16	2
Total	1281	52

**Table 3:** Inclusion and exclusion criteria.

Inclusion Criteria	Exclusion Criteria
Studies that focused on PRWs.	Studies that were not written in English.
Studies that reported different signaling mechanisms in healthcare.	Excluded papers other than journal articles or conference proceedings.
Studies that analyzed patients’ choice or patient decision-making process.	Remove duplicate/similar studies by maintaining the most comprehensive and current version.
Studies that analyzed patients’ opinions as online physician reviews.	Studies without any practical, theoretical, or statistical evidence were excluded.
Studies with clear aims/objectives.	
Studies that addressed and described the research context properly.	
The findings of the studies were in line with our research purpose.	
Studies that were peer-reviewed and written in English.	
Studies that were qualitative, quantitative or mixed-methods, in nature.	

**Table 4:** Criteria for evaluating the quality of studies.

Criteria	Response Score	Score Obtained
Is the study aim/objective clear?	Yes = 1/moderately = 0.5/no = 0	31 studies 88%
Is the research context dealt with well?	Yes = 1/moderately = 0.5/no = 0	21 studies 92%
Based on the research findings, what percentage is the quality rate acceptance?	>80% = 1/under 20% = 0/between = 0.5	

**Table 5:** Quality scores of included papers.

Quality Scores						
	Poor (<26%)	Fair (26–45%)	Good (46–65%)	Very Good (66–85%)	Excellent	Total
Number of articles	3	1	11	17	20	52
Percentage of articles	5.76	1.92	21.15	32.69	38.46	100

## Data Availability

Data available upon request from the corresponding author.

## Appendix A

**Table A1:** Summary of e-health literature from 2010 to 2020 on physician rating websites and patient choice regarding health consultation.

Reference	Data Collection	Variables	Research Method	Findings
Wu [52]	Web scraping	Individual literacy social support Information quality Service quality	SEM-PLS	Information quality is significantly associated with patient satisfaction.
Jin, Yan, Li and Li [71]	Web scraping	Information quality	Text mining Logistic regression	Higher information quality leads to information adoption.
Storino, Castillo-Angeles, Watkins, Vargas, Mancias, Bullock, Demirjian, Moser, and Kent [116]	Collection of 50 pancreatic cancer websites	Information usefulness	Assessment by expert panel	Online information on pancreatic cancer lacks effective information about alternative therapy.
Zhang, Guo, Lai, and Yi [82]	Web crawling	Interpersonal unfairness Informational unfairness	Logistic regression	Informational unfairness contributes to the development of a quality relationship.
Li, Tang, Yen David, and Liu [38]	Web crawling	Online reputation Offline status Online self-representation	Regression analysis	Online reputation, offline status, and online self-representation are also positively related to a physician’s online order volume.
Yang, Guo, Wu, and Ju [47]	Web crawling	Patient-generated information System-generated information	Regression analysis	Positive system and patient-generated information will positively affect patients’ decisions.
Shah, Yan, Shah, Shah, and Mamirkulova [5]	Web crawling	EWOM Physician trustworthiness	OLS	EWOM and trustworthiness significantly influence physician’s economic returns.
Cao, Liu, Zhu, Hu, and Chen [30]	Web crawling	Service quality EWOM	OLS	Physician’s service quality and eWOM would affect patients’ selection decision.
Li, Tang, Jiang, Yen, and Liu [49]	Web crawling	Online knowledge Contribution Online reputation	Regression analysis	Knowledge contribution and reputation are positively related to a physician’s online income.
Deng, Hong, Zhang, Evans, and Chen [32]	Web crawling	Online effort Online reputation	OLS	Patients prefer to choose online physicians with greater effort and reputation.
Wu and Lu [45]	Web crawling	Online reputation Offline reputation Online services	OLS	Impact of online popularity is positive on online booking service in hospitals.
Yang and Zhang [48]	Web crawling	Free feedback Paid feedback	OLS GLS	Paid feedback has a greater effect on patient choice than free feedback.
Liu, Guo, Wu, and Wu [28]	Web crawling	Offline reputation Online reputation	HLM	Physician’s reputation and hospital’s reputation have a positive effect on the number of appointments.
Chen, Yan, and Zhang [108]	Web crawling	Physician’s login behavior	Regression analysis	Physician login behavior positively influences patient choice.
Han, Qu, and Zhang [104]	Questionnaire	Neighbor-recommended physician Trust in OPRs Review valence	Partial correlation analysis	Patients with high-risk diseases would be more likely to select a neighbor-recommended physician who has positive reviews than patients with low-risk diseases.
Luo, Chen, Wu, and Li [109]	Web crawling	Physician’s colleague multi-channel access (SI), ratings	OLS regression	SI and patients’ rating significantly and positively affect multi-channel access in an OHC.
Wu and Lu [44]	Web crawling	Colleague reputation Physician reputation	Fractional logistic regression	There is significant impact of focal physician’s colleagues’ reputation and focal physician reputation on the patients’ odds of posting treatment experience.
Guo, Guo, Fang, and Vogel [60]	Web crawling	Status capital Decisional capital	OLS	The social and economic returns of doctors in OHCs are positively associated with their status and decisional capital.
Shan, Wang, Luan, and Tang [106]	Eye-tracking experiment Surveys	Cognitive trust Affective trust	PLS-SEM	Cognitive trust and affective trust both positively affected patients’ choice of physician.
Yang, Diao, and Kiang [110]	Web crawling	Ability Reputation Benevolence	Logarithmiclinearregression	Physician’s trustworthiness (ability, reputation, and benevolence) has positive impact on a physician’s sales.
Chen, Rai, and Guo [31]	Web crawling	Credibility Benevolence	Regression analysis	Volume of reviews significantly moderates the impact of credibility on popularity, while valence and variance significantly moderate the influence of benevolence on online popularity and price premium.
Guo, Guo, Zhang, and Vogel [61]	Web crawling	Weak ties Strong ties	Smart-PLS	Significant effect of D–P tie strength was found on doctors’ returns in the online healthcare context.
Wu and Lu [65]	Web crawling	Pricing and quantity of online services	OLS	Service quantity positively impacts patient satisfaction.
Li, Zhang, Ma, and Liu [50]	Web crawling	Online reputation Online self-representation	Regression analysis	A market served by many doctors with strong online reputations or high levels ofself-representation will be less concentrated.
Zhang, Guo, and Wu [54]	Web crawling	Online contributions Quantity of online contributions (popularity), Quality of online contributions (reputation)	Regression analysis	The relationship between quantity of online free service contributions and doctor’s private benefits was found to be positive.
Wu and Deng [53]	Web crawling	Specification Credibility Coordination	OLS regression	Physician’s specification, credibility, and coordination are positively related to order quantity.
Liu, Guo, Wu, and Vogel [51]	Web crawling	Online doctor efforts	OLS and hierarchical regression	Breadth and depth of online doctor efforts are associated with doctor reputation and popularity.
Yang, Guo, and Wu [46]	Web crawling	Response speed Interaction frequency	Regression analysis	Physician’s response speed and interaction frequency would significantly affect patient satisfaction.
Lu and Wu [63]	Web crawling	Overall review rating Number of reviews	OLS regression	The rise in ratings and number of reviews will result in increase in the number of outpatient visits.
Zhao, Li, and Wu [56]	Web crawling	External WOM Peer influence Internal WOMA ppointment quantity	Three-stage least square	The number of doctors’ votes, followers, and reviews (external WOM), peer influence, internal WOM, and appointment quantity significantly influence patients’ doctor choice.
Liu, Zhou, and Wu [111]	Web crawling	Title, satisfaction, review volume, service attitude, technical level, clarity of explanation, ethics	Negative binomial regression	All variables significantly influence online appointment services received by the focal doctor except for service process.
Lu and Wu [40]	Web crawling	Technical quality Functional quality	OLS	Patients make appointments with physicians with technical and functional quality.
Lu and Zhang [73]	Questionnaire	Physician–patient communication Information quality Decision-making preference Physician–patient concordance	Smart-PLS	Physician–patient communication in OHCs positively impacts patient compliance through mediations of the perceived quality of internet health information, decision-making preference, and physician–patient concordance.
Emmert, Meier, Pisch, and Sander [115]	Questionnaire	–	Interviews	–
Laugesen, Hassanein, and Yuan [78]	Questionnaire	Perceived information asymmetry Patient–physician concordance	Smart-PLS	Perceived information asymmetry and patient–physician concordance significantly influence patient compliance for health consultation.
Chen, Guo, Wu, and Ju [95]	Web crawling	Patient activeness Informational support Emotional support Patient satisfaction	Text miningRegression analysis	Patient activeness has significant impact on informational and emotional support. While informational and emotional support significantly influence patient satisfaction.
Yang, Du, He, and Qiao [29]	Web crawling	ReputationMonetary rewardD–P interaction	Regression analysis	Reputation, monetary reward, and D–P interaction significantly influence physician contribution to OHC.
Chen, Baird, and Straub [81]	Web crawling	Affective signals Informational signals Informational support Social support	Text mining Regression analysis	Affective signals and informational signals significantly influence informational support and social support from OHCs.
Khurana, Qiu, and Kumar [14]	Web crawling	Doctor response to patient questions	Regression analysis	Doctor response significantly influences user perception of medical services offered.
Greenwood, Agarwal, Agarwal, and Gopal [99]	Web crawling	Individual expertise Organizational expertise Adoption of new practices	Econometric analysis	There is a significant influence of individual and organizational expertise on physician behavior.
Shah, Yan, Khan, and Shah [67]	Web crawling	Review-related signals Service-related signals	Text mining Regression analysis	Review-related signals and service-related signals significantly influence patients’ behavior.
Hong, Liang, Radcliff, Wigfall, and Street [15]	Systematic review	–	Content analysis	–
Hao and Zhang [33]	Web crawling	–	Topic modeling	–
Hao, Zhang, Wang, and Gao [34]	Web crawling	–	Topic modeling	–
Li, Liu, Li, Liu, and Liu [37]	Web crawling	–	Content analysisTopic modeling	–
James, Villacis Calderon, and Cook [35]	Web crawling	–	Topic modeling	–
Jung, Hur, Jung, and Kim [36]	Web crawling	–	Topic modeling	–
Wallace, Paul, Sarkar, Trikalinos, and Dredze [4]	Web crawling	–	Topic modeling	–
Zhang, Deng, Hong, Evans, Ma, and Zhang [7]	Web crawling	–	Content analysis	–
Bidmon, Elshiewy, Terlutter, and Boztug [3]	Survey	–	Regression analysis	–
Pang and Liu [112]	Web crawling	–	Topic modelingQualitative analysis	–
Noteboom and Al-Ramahi [114]	Web crawling	–	Topic modeling	–
